# Impact of Communication Measures Implemented During a School Tuberculosis Outbreak on Risk Perception among Parents and School Staff, Italy, 2019

**DOI:** 10.3390/ijerph17030911

**Published:** 2020-02-01

**Authors:** Davide Gentili, Andrea Bardin, Elisa Ros, Cinzia Piovesan, Mauro Ramigni, Maria Dalmanzio, Marco Dettori, Antonietta Filia, Sandro Cinquetti

**Affiliations:** 1 Public Health Office, Local Health Unit 2 Marca Trevigiana, 31100 Treviso, Italy; elisa.ros@aulss2.veneto.it (E.R.); maria.dalmanzio@aulss2.veneto.it (M.D.); sandro.cinquetti@aulss2.veneto.it (S.C.); 2 Department of Medical, Surgical and Experimental Sciences, University of Sassari, 07100 Sassari, Italy; madettori@uniss.it; 3 Department of Cardiac, Thoracic and Vascular Sciences, Public Health Unit, University of Padua, 35100 Padua, Italy; andrea.bardin@aulss2.veneto.it; 4 Epidemiology Office, Local Health Unit 2 Marca Trevigiana, 31100 Treviso, Italy; cinzia.piovesan@aulss2.veneto.it (C.P.); mauro.ramigni@aulss2.veneto.it (M.R.); 5 Department of Infectious Diseases, National Health Institute (Istituto Superiore di Sanità), 00100 Rome, Italy; antonietta.filia@iss.it

**Keywords:** tuberculosis, school outbreak, risk communication, crisis communication, community outrage, outrage management, emotional epidemiology

## Abstract

Risk perception has a significant impact on decisions people make when facing a threat: a mismatch between actual hazard and perceived risk can lead to inappropriate behaviours and suboptimal compliance to recommended public health measures. The present study was conducted in the aftermath of a tuberculosis (TB) outbreak that occurred in 2019 in a primary school in Italy. The aim was to evaluate the impact of communication measures implemented by local health authorities (including face-to-face meetings between LHAs and the local population, weekly press announcements, implementation of a telephone hotline and of an information desk, and social media communication), on risk perception among parents of schoolchildren and school staff, and to identify factors related to a change in risk perception before and after the said activities. An anonymous questionnaire was administered to parents of schoolchildren (*n* = 846) and to school staff (*n* = 70). Participants were asked about the level of risk they had perceived at two distinct times: when they first became aware of the outbreak and following implementation of communication activities. A significant reduction of perceived risk was found in both groups (*p* < 0.001) following the communication activities. The largest reduction was found among participants who reported having appreciated the meetings with the LHA healthcare staff. Our findings suggest that keeping an open approach, explaining the actual threat to the population and adapting communication to different listening skills, are essential for health authorities to successfully manage a public health emergency.

## 1. Introduction

Risk communication represents an integral component of an effective outbreak response. The actual implications of a public health threat may not be immediately understood by the population and when an individual’s risk perception of a threat does not match the actual hazard posed by the threat, personal choices and actions taken may not be completely effective and compliance to recommended public health measures may be inadequate. In the case of an infectious disease outbreak, effective communication with the population can improve adherence to recommended public health measures, and improve efficiency in a situation where human resources are called upon to make extraordinary efforts [1].

In critical situations, people take in information, process it, and act on it in a different way than they would normally. Previous research has shown that the perception of risk has a significant impact on the actions that people take when facing a threat [2,3,4]. The Centers for Disease Control and Prevention (CDC) field epidemiology manual highlights how persons perceive risks differently, depending on how likely they think the actual hazard will affect them personally, and their beliefs about how severe the harm might be. According to the CDC, persons accept risks more easily or feel less outrage when the risk is voluntary, under their control, has clear benefits, is naturally occurring, is generated from a trusted source, or is familiar. By contrast, risk is perceived as less acceptable when it is imposed on the affected population, not allowing any choice, is controlled by others, has intangible or deferred benefits, is human-made, generated by an untrusted source, is new or exotic and affects primarily children [5]. For instance, an outbreak of an infectious disease for which there is pre-existing information about its infectiousness and mode of transmission will lead to a lower perception of risk among the public than an outbreak of a disease that is not scientifically understood, even when the known disease poses a greater health threat. In his 1993 book “Responding to Community Outrage” Peter Sandman claims that experts face two core communication tasks in a risk controversy: the need to talk better (e.g., to explain the actual magnitude of the hazard) and the need to listen better (e.g., to acknowledge when the outrage is high and take action to reduce it). Whereas the first task is usually acknowledged by everyone, the second task tends to be ignored rather frequently. According to Sandman, in order to attain the best infectious disease outbreak response, the outrage should be proportional to the hazard; in practice, however, often it is difficult for the public to comprehend and evaluate the threat posed by a disease accurately [6,7].

### Theoretical Background

All communities need a way to communicate about present, emerging, and evolving risks: public health hazards respect no boundaries in a world where people and disease travel faster and further than at any other time in human history. The International Health Regulations (IHR) have highlighted risk communication as one of the eight core capacities that World Health Organization (WHO) member states need to build and sustain as part of a global agreement to detect and respond to public health threats. In 1992, Covello V. T. defined risk communication as the “process of exchanging information among interested parties about the nature, magnitude, significance, or control of a risk. Interested parties include government agencies, corporations or industry groups, unions, the communications media, scientists, professional organizations, public interest groups, communities, and individual citizens” [8]. Similar definitions have been proposed more recently, which emphasize the importance of risk management (McComas K. A, 2006), the need for dialogue between communicators and stakeholders (Palenchar M. J, 2005), and the necessity of ongoing risk monitoring (Coombs W. T, 2012) [9,10,11].

Palenchar M.J specifically states that: “communication objectives are not just to gain additional support for the risk-generating organization or allay the concerns of community residents and other involved individuals and parties, but rather a constructive dialogue that legitimately addresses risk assessment, abatement, policy and communication” [10].

The interest in how risk communication could be most effective has increased over time, with initial questions focused on message creation, and expanding to the way audiences process information and act on it, leading to a deep body of risk communication research. 

Fischhoff B. was one of the leading pioneers of risk communication research; his work culminated in the identification of seven evolutionary stages of risk communication and best practices: get the numbers right, tell the public the numbers, explain what the numbers mean, show people they have accepted similar risks before, explain that the benefits of public health measures outweigh their costs, treat people with respect, make people partners with risk communicators [12].

Since Fischhoff’s seminal work, authors have investigated additional factors that can be crucial for effective public risk communication and guidelines have been outlined by leading reference organizations such as the WHO and CDC.The crisis and emergency risk communication model (CERC) of CDC, as well as the WHO best practice guidelines for communicating with the public during an outbreak, suggest that, in managing a public health crisis, public health authorities should focus on effective communication with the population in order to reduce their psychological burden and help making them feel empowered to take the best actions for themselves [1,13]. 

Understanding how people take in information during a crisis is essential for better communication planning. One of the main problems that arise during stressful events is that people cannot fully process information correctly, memory capacity is impaired, and over-elaborate communication can easily be misinterpreted. An important suggestion is, therefore, to deliver simple communication messages [13].

In public health, the communication process around a crisis involves the collection, processing, and dissemination of information required to address the crisis and is divided into three main phases: pre-crisis, crisis and post-crisis [14]. 

In the “crisis” phase, the main processes involve the collection and processing of information for crisis team decision making and the creation and dissemination of crisis messages. During this phase, it can be difficult for health professionals to convince people to do something that seems counterintuitive to them, since people can hold a number of strong but incorrect beliefs. In addition, different experts can offer contrasting views on complex subjects, thus increasing uncertainty, fear and distrust among people which, in turn, can worsen their compliance with control measures. To address this problem, it is essential that messages come from a credible source. Identifying experts and institutions that are highly regarded in the community, and involving them in the decision-making process is a good strategy to ensure that public health actions are more widely accepted, contextually appropriate and that communication is community-owned [13]. A multidisciplinary response team must be clearly identified to take evidence-based decisions, share information and adopt the best counter-measures to the problem. Trust is the foundation of effective communication and can be built through three basic elements: transparency, accountability and listening [1,13]. Transparency implies that communicators must inform the community-clearly and early on-about what they do and do not know, and about what they are doing. It is essential not to hide relevant information. Accountability implies that communicators demonstrate that they and their managers are responsible for what is being done, said and promised. Finally, listening implies that communicators must show clear awareness of the public’s concerns. In practice, this means monitoring the media, and using other methods to understand changing public opinions about the risks posed by an outbreak and the effectiveness of its management.

In addition, in planning a communication strategy during a crisis, public health authorities must take into account that people tend to look for messages to be confirmed before acting on them, and often rely on multiple information sources before making a choice, including social media networks to see what their contacts are saying. Moreover, people tend to turn to a known and credible local leader for advice [15,16]. All of this makes it even more crucial for healthcare authorities to use consistent messages during a crisis and communicate them as soon as possible. The speed of a response can also be a critical factor in reducing harm in crisis scenarios, making it sometimes necessary that more accurate information be delivered later on [1,13].

Based on the above premises, we conducted a study to evaluate the impact of communication measures implemented by local public health authorities during the management of a school tuberculosis outbreak, on perception of risk in the population involved in the outbreak. To this end, we compared the level of risk perception among children’s parents and school staff during two different phases of the outbreak: at the time when they first became aware of the outbreak and after communication measures had been implemented. We also aimed to identify individual factors that were related to a change in risk perception between the two phases.

## 2. Materials and Methods

### 2.1. Study Setting and Description of the Outbreak

The present study was conducted during a tuberculosis outbreak that occurred in early 2019 in a primary school in north-eastern Italy [17]. The index case of the outbreak, a 10-year-old child, was notified to the local health authorities (LHA) on 5 March 2019. A timely source case investigation was conducted, by testing close contacts, leading to the identification, on 8 March 2019, of the primary case, a schoolteacher in the same school attended by the index case. Thirteen active tuberculosis cases and 50 latent tuberculosis infections (LTBI) were recorded among tested subjects.

Since the local population was not familiar with tuberculosis, due to the low incidence of the disease in Italy (6.5 cases/100,000), and the risk was perceived to be out of a person’s control, the outrage related to this event was expected to be high, with the potential to cause a communication crisis scenario.

### 2.2. Response by Local Health Authorities

To respond to the emergency with the greatest possible efficiency, an outbreak management team, coordinated by the Public Health Department of the Local Health Unit was formed, with the support of specialists in pediatrics, microbiology, infectious diseases and radiology. The team took shared decisions, in keeping with national guidelines and international recommendations on tuberculosis [18,19,20]. The Department of Infectious Diseases of the Italian National Institute of Health (ISS) was also involved and provided support and technical expertise during each phase of the epidemiological investigation. In total, more than 1100 Mantoux tests were performed: 691 subjects were tested soon after the discovery of the index and primary case, of whom 493 were retested 10 weeks later according to guidelines. The compliance rate to the retesting was 100%, meaning that there were no drop out to the planned follow up. The management of the communication with people and the media represented a challenge in itself.

The communication measures had, on the one hand, to manage the excessive outrage observed, not proportionate to the actual risks, and on the other hand, avoid minimizing a real threat that could have caused many more cases in excess of those already notified. 

During the outbreak, LHA received numerous inappropriate requests, including demands to close down the school, perform mass vaccinations, and to exclude children from school solely on the basis of a positive Mantoux test result. This was a clear sign that the outrage level was not proportionate to the level of actual risk and that an ad hoc communication strategy was required. Thus, a communication intervention was developed, based on openness, transparency and wide availability towards the public, in line with the WHO’s Outbreak Communication Planning Guide [21] and CDC’s CERC model [13]. The intervention included a number of communication activities: two face-to-face meetings were held in the school between task force members and the local population, to provide clear information and to address questions and doubts; press announcements were released every week and after each new diagnosed case, in order to keep the population updated about the progress of the epidemiological investigation underway; a telephone hotline was activated on weekdays from 2.00 pm to 4.00 pm to answer questions by the public (a total of more than 100 calls were managed from 11 March to 10 June 2019); an information desk with trained staff of the Local Health Authority (LHA) was set up on weekdays from 8.00 am to 9.00 am in a dedicated room inside the school, to facilitate access by pupils’ parents; and finally, communication via social media, with the sharing of official press releases, was provided through the LHA’s official Facebook page. Social media, such as Facebook, allow users to comment onposts. To highlight potential information and communication gaps among the population, the LHA team analyzed and evaluated each comment to official posts on the LHA’s Facebook page.

Questions asked by the public included, among other things, the infectiousness of subjects with a positive Mantoux test result and the need for isolation measures, the sanitation of the school building (especially the gym), the risk of infection in places other than the school (e.g., the school bus), the availability of a tuberculosis vaccine.

### 2.3. Population and Study Design

Our study population consisted of school staff and (both) parents of schoolchildren who had initially tested negative to tuberculin (Mantoux) skin testing and who were undergoing retesting 10 weeks later, as per recommended guidelines. Parents of children diagnosed with active or latent tuberculosis at initial testing, and school staff with the same conditions, were excluded from the study. An anonymous questionnaire was administered to study subjects.

The questionnaire included a total of 14 questions, with sections about responders’ demographic information, degree of exposure to the primary case and knowledge about tuberculosis. Two questions (numbers 8 and 9 in Table 1 and Table 2) investigated the level of perceived risk at two distinct times: when they first became aware of the outbreak (before the intervention by LHA) and the current level, after the intervention by the LHA, hereafter referred as T0 and T1 respectively. Moreover, it investigated if meetings were considered clear enough and, if not, which aspects were found to be unsatisfactory. Furthermore, it investigated the timeliness of the intervention and which were the main sources consulted to gather information about tuberculosis and its possible consequences.

The individual factors that were considered in their relation to a change in risk perception between T0 and T1 were: hours per week the schoolchildren and school staff spent with the primary tuberculosis case, knowledge of tuberculosis as a disease before the event occurred at school, awareness of tuberculosis cases in the province of residence in the past two years, timeliness of the intervention carried out by LHA, usefulness of the intervention in clearly defining the situation, aspects of the tuberculosis outbreak that caused the greatest concern in the subjects (the number of notified cases, the type of disease, the fact the disease happened at school, the fear of being affected, other fears) and which sources of information had been used to find out about the disease and its possible consequences (institutional websites, specialized websites, social networks, TV and newspaper, healthcare staff of the LHA, general practitioner and pediatrician, other healthcare professional, other people).

The complete questionnaire is found in Supplementary S1.

Two copies of the questionnaire were distributed to each family and each parent was asked to complete one independently and return it in a closed envelope within three days.

### 2.4. Statistical Analysis

Responses by parents and school staff were stored separately using Microsoft Excel (Microsoft Corporation, Redmond, DC, USA) and analysed with R statistical software (R Development Core Team, Vienna, Austria). A Wilcoxon signed-rank test was used to measure the change in risk perception among parents and school staff, between T0 and after T1 Possible answers about the perceived risk included four ordinal levels: no risk, low, moderate and high risk. We measured the number and the proportion of respondents who reported each of the risk levels, comparing T0 and T1. Moreover, we divided our study population into four subgroups, one for each risk level indicated at T0; then we evaluated the risk perception at T1 for each subgroup.

In order to evaluate the factors related to changes in the perceived level of risk, we carried out a logistic regression analysis, with the creation of the binary variable “post-intervention risk perception” indicating the presence/absence of decreased risk perception after the communication intervention. The variables included in our model were: perceived risk level before the intervention, (reported) usefulness of meetings with the healthcare staff of the Local Health Authority in clearly defining the situation, use of websites, newspapers, television and social networks as primary sources of information on tuberculosis, fear of being infected, hours of exposure to the primary case, age, sex, and level of education. We then stratified by initial perceived risk level. A model restricted to the moderate-high initial perceived risk strata was finally obtained using a stepwise process.

The logistic regression analysis was carried out exclusively among parents due to an insufficient number of school staff to reliably evaluate a possible relationship of the independent variables to the dependent variable “Post-intervention risk perception”.

## 3. Results

A total of 916 questionnaires were distributed (846 to parents and 70 to school staff), with a response rate of 89.8% among parents (760 completed questionnaires received) and 90,0% among school staff members (63 completed questionnaires). Table 1 and Table 2 report the responses given by parents and school staff, respectively, including demographic characteristics.

Over 75% of parents reported that they had knowledge of tuberculosis before the school outbreak, but only 13.8% were aware of the occurrence of cases in their province of residence in the last two years. A similar result was found among school staff, with 87.3% reporting that they knew about TB but only 30.2% were aware of cases in the area in the last two years.

**Figure 1:**
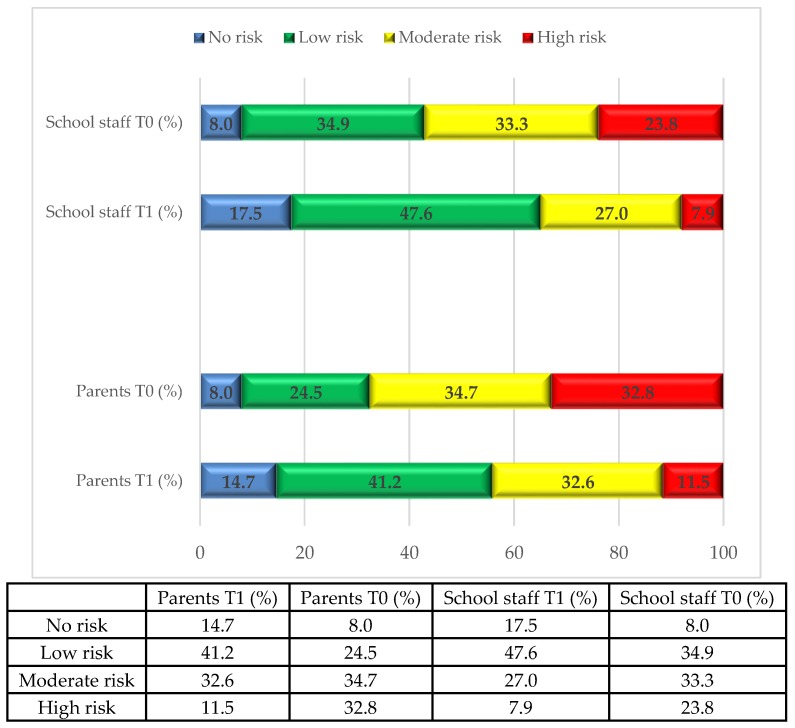
Changes in risk perception before (T0) and after (T1) intervention by local health authority (*n* = 756 parents and 63 school staff).

**Figure 2:**
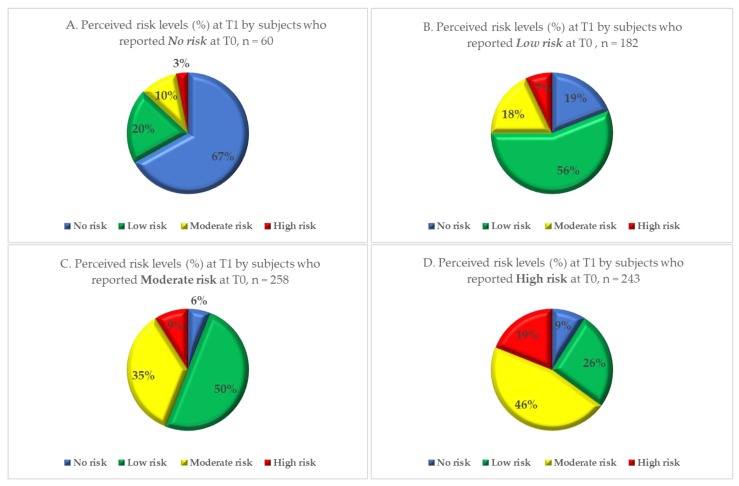
Level of perceived risk after among parents (*n* = 743) with no (Quadrant. **A**), low (Quadrant. **B**), moderate (Quadrant. **C**) and high (Quadrant. **D**) perceived risk at baseline, after the local health authority intervention.

**Figure 3:**
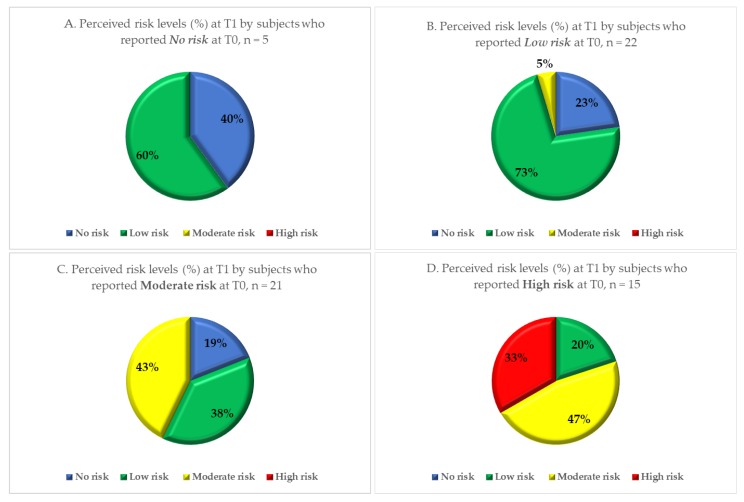
Level of perceived risk after among school staff (*n* = 63) with no (Quadrant. **A**), low (Quadrant. **B**), moderate (Quadrant. **C**) and high (Quadrant. **D**) perceived risk at baseline, after the local health authority intervention.

Over 66% of parents and 57.1% of school staff perceived a moderate to high level of risk before the intervention (T0); but only 43.4% and 34.9% respectively did so after the intervention (T1). In particular, the proportion of parents reporting a high level of perceived risk dropped from 32.4% to 11.3% and the proportion of school staff decreased from 23.8% to 7.9%. Thirteen percent of subjects reported no perceived risk at T0, and 25.0% of subjects reported a low level of risk at T1 showed an increase in perceived level of risk at T1 (to a moderate or a high level). The proportion of subjects reporting each of the four levels of perceived risk before (T0) and after (T1) the local health authority intervention is shown in Figure 1. The variation in perceived risk in each risk subgroup is shown in Figure 2 for the parents and Figure 3 for the school staff.

A comparison between risk perception before and after the intervention (Wilcoxon signed-rank test) showed a significant reduction in perceived level of risk in both the parent (*p* < 0.001) and school staff groups (*p* < 0.001). The Wilcoxon test results are shown in the Supplementary Material S2 and S3.

Nearly 80% of parents thought the intervention was timely, and 75.3% that the meetings with the healthcare staff of the LHA were useful in clearly defining the situation. These proportions are even higher among school staff: over 90% reported that they considered the intervention by the LHA to be timely and 84.1% that the explanations given were useful.

Among the 176 parents (23.1% of responders) who reported that the intervention by LHA was not useful in clearly defining the situation, a majority motivated their reply by specifying that “*conflicting explanations were given*” 109 (61.9%). Moreover, 93 (52.8%) reported “*the risk was underestimated”* and 80 (45.5%) that “*communication was not transparent”*. Similar results emerged from the school staff survey, with 6 of 9 who reported that the intervention was not useful (66.7%) reporting that conflicting explanations were given, 7 of 9 (77.8%) that communication was not transparent, and 22.2% (2 people) that risk was underestimated (more than one answer was possible).

When asked about their greatest concerns regarding the outbreak, 44.7% of parents pointed to the fact that it happened at school and 44.1% to the high number of notified cases. Other major concerns were the fear that their child may be infected (36.2%) and the type of disease (29.3%). Among school staff, the greatest concerns regarded the high number of notified cases of TB (61.9%), the school setting (25.4%), the fear of being affected (22.2%) and the type of disease (15.9%).

Among the information sources used by parents to find out more about the disease and its possible consequences, 52.4% relied on institutional websites (Ministry of Health, Epicentro website of the National Health Institute), 35.5% relied primarily on LHA healthcare staff, 25.3% on general practitioners or paediatricians, 16.7% on television reports and newspaper articles, 15.7% on web searches in specialized sites and 5.8% on social networks. School staff considered the healthcare staff of the LHA as the predominant information source on TB and its possible consequences, followed by general practitioners and paediatricians.

To evaluate which of the factors investigated by the questionnaire had a significant influence on the dependent variables “Post-intervention risk perception” among parents who perceived a moderate and high risk at T0, a logistic regression was performed (Table 3).

Among the parents who perceived a moderate and high risk at T0, having perceived the meetings as useful in clearly defining the situation was associated with a reduction in perceived risk at T1 (OR 2.08, CI 1.37, 3.17). Parents’ educational level influenced the degree to which perceived risk decreased from T0 to T1 as well: the odds ratio (OR) among the lowest level of education was 0.12 (CI, 0.02,0.6) compared to the group with the highest level of education. Furthermore, using social networks as a major source of information about the disease and its possible consequences was also associated with a reduction in perceived risk at T1, although not reaching statistical significance. Finally, gender was not found to influence the possibility of a reduction in risk perception

## 4. Discussion

This study evaluated the impact of a communication strategy implemented during a school tuberculosis outbreak on risk perception by parents and school staff. The intervention aimed to provide effective and useful communication with the public and the media, based on a transparent and open approach, and included the arrangement of organized meetings, a call centre and an information desk. The study also investigated the factors related to changes in level of perceived risk, before and after the intervention. To our knowledge, no similar studies performing this type of direct evaluation have been previously conducted.

Our results suggest that a comprehensive communication strategy by public health services, focused on openness and availability, can help in reducing a population’s outrage during a crisis communication scenario.

A significant reduction of perceived risk was observed after the communication intervention, among participants who reported a moderate/high initial level of perceived risk. Moreover, among those reporting a low/absent initial level of perceived risk, the risk level after the intervention did not increase significantly. The reduction in perceived risk reported by participants who found meetings with LHA useful in clearly defining the situation suggests that face-to-face meetings can play an important role in establishing effective communication with the public, allowing for a bidirectional exchange. A communication approach aimed at building credibility and trust, focused on empathy, openness, speed of release and accuracy of information, has been also suggested by the Crisis and Emergency Risk Communication model (CERC) by CDC [13,22,23]. A similar communication approach was also implemented in studies carried out during recent Ebola epidemics in central and west Africa [24,25].

Our analysis did not specifically investigate the impact of the LHA’s Facebook page, but it focused, more generically, on the use of social networks as the main source of information about the disease. However, besides traditional media sources, any news concerning the outbreak was shared in real time on the LHA’s official Facebook page, meaning that this was the fastest and most reliable source of information available on the internet. We found an association, at a confidence level of 90%, between use of social media as the main source of information and a reduction in risk perception, although it did not reach statistical significance at a 95% confidence level. These results seem to be in agreement with what has been previously observed on the usefulness of social media by public health authorities in an emergency scenario. A systematic review of literature [26] on social media and outbreaks of emerging infectious diseases has, in fact, shown that social media is an effective mean of communication during outbreaks, especially to deliver essential and updated medical information to the population [15]. Social media have been shown to be instrumental in informing the population about recent emerging infectious disease (EID) outbreaks such as the Ebola outbreak in 2014 and the H1N1 outbreak in 2009 [27]. The WHO itself calls for a more proactive use of social media by public health authorities to share health messages with journalists, physicians, and the general public, especially for their potential effectiveness in countering misinformation about EID [28].

The fact that the vast majority of study participants (nearly 80% of parents and over 90% of school staff) reported that the communication measures by the LHA had been implemented in a timely manner can be seen as a further sign of the positive impact the intervention had on the population.

Despite the overall reduction in risk perception achieved, 23% of parents reported that the two face-to-face meetings had not been useful in clearly defining the situation. A possible explanation for this lies in the fact that, during the first meeting, LHA staff placed much emphasis on the relatively low infectiousness of children with active tuberculosis. Following this meeting, the number of cases identified in the contact investigation had increased, possibly leading to the perception that the previous communication had been misleading.

Although the school setting is far from being a risk-free environment, especially because of the closeness of contacts between numerous individuals in restricted spaces and the lengthy exposure time, risk perception by the population is generally that of a relatively safe environment. Our results show that the setting and the high number of cases were the main causes of parental concern, suggesting that the risk of occurrence of such an outbreak was considered substantially irrelevant prior to this event.

This study involved a large sample, representative of the outbreak event studied, and a high response rate was achieved. A close deadline was given to complete the questionnaire, making it unlikely that participants were influenced by the responses given by the other participants. All questionnaires were collected and elaborated maintaining anonymity of study participants, increasing the likelihood that responses were truthful.

The main limitation of this study consists in possible confounding factors in the relationship between the reduction of perceived risk among study participants and the public health intervention. It is possible that the reduction in perceived risk observed was influenced by different factors other than the public health intervention itself. For instance, between T0 and T1 all school children that had been in contact with the primary case had received a first Mantoux test and the vast majority had a negative result. This in itself may have led to lowering the perceived risk in some participants. Moreover, between the two moments, all children and staff with active TB were no longer attending the school, eliminating a possible outrage factor.

Another main limitation is that the questionnaire was administered at a single moment in time (T1) so the perceived risk at T0 was evaluated only retrospectively. This could make its report prone to recall bias. However, T0 was defined as the moment parents and school staff first learned of the outbreak, a specific moment whose emotional impact is not easy to forget.

This study highlights the importance for health authorities to acquire knowledge about risk perception among the population during an epidemic event and, therefore, to assess the population outrage, so as to design a more effective public health response [29,30].

In particular, it suggests that explaining the actual threat to the population and adapting communication strategies to different listening skills is essential for a successful public health intervention, as also pointed out by the European Centre for Disease Prevention and Control (ECDC) in a literature review on effective risk communication for the prevention and control of communicable diseases [31]. Our finding that the implemented communication initiatives were less effective in individuals with a lower education level deserves to be investigated further, to enable policy makers and public health experts to better design more targeted measures.

## 5. Conclusions

Ours findings suggest that a comprehensive communication strategy by public health services, focused on openness and availability, can help in reducing a population’s risk perception during a major public health emergency with high public outrage potential.

Explaining the actual threat to the population and adapting communication strategies to different listening skills, seems essential for health authorities to successfully manage a public health emergency. In particular, allowing for a bidirectional exchange, as can be done through phone calls and direct meetings, can play an important role in establishing effective communication with the public during a major public health crisis. Further studies may strengthen our findings and help to better delineate effective communication measures to deal with public outrage during major public health emergencies.

## Figures and Tables

**Table 1:** Responses given by parents, risk perception questionnaire *n*= 760.

Number	Questions	Answers	*n*	%
1	Gender	Female	403	53.0
		Male	357	47.0
2	Age	≤30 yrs	31	4.1
		31–40 yrs	259	34.1
		41–50 yrs	415	54.7
		>50 yrs	53	7.0
		Missing	1	0.1
3	Educational level	Primary school	21	2.8
		Lower secondary school	138	18.1
		Upper secondary school	435	57.2
		Higher education (university degree and Ph.D.)	164	21.6
		Missing	2	0.3
4	Which grade does your child attend?	1st grade	163	21.4
		2nd grade	148	19.5
		3rd grade	150	19.7
		4th grade	145	19.1
		5th grade	165	21.7
		Missing	2	0.3
5	How many hours per week did your child spend in close contact with the primary tuberculosis case?	None	533	70.1
		<1	87	11.4
		1–5	29	3.8
		6–10	41	5.4
		>10	48	6.3
		Missing	22	2.9
6	Did you know about tuberculosis as a disease before the recent events occurred at school?	Yes	575	75.7
		No	184	24.2
		Missing	1	0.1
7	Were you aware of tuberculosis cases in your province of residence in the last 2 years?	Yes	105	13.8
		No	647	85.1
		Missing	8	1.1
8	What was your perception of risk when you became aware of these tuberculosis cases?	No risk	60	7.9
		Low risk	184	24.2
		Moderate risk	261	34.3
		High risk	246	32.4
		Missing	9	1.2
9	What is your current perception of risk also considering the intervention carried out by the local health authority (LHA)?	No risk	110	14.5
		Low risk	308	40.5
		Moderate risk	244	32.1
		High risk	86	11.3
		Missing	12	1.6
10	Do you think the intervention of the LHA following notification of the first case of tuberculosis was timely?	Yes	607	79.9
		No	132	17.4
		Missing	21	2.7
11	Were the meetings with the LHA healthcare staff useful in clearly defining the situation?	Yes	572	75.3
		No	176	23.1
		Missing	12	1.6
11.1	If you answered No to the previous question, what do you think was missing?	I wanted more meetings	19	11
		Conflicting explanations were given	109	61.9
		The meetings were delayed	58	33
		Communication was not transparent	80	46
		The risk was underestimated	93	52.8
		Other	15	8.5
12	What aspect of the current tuberculosis outbreak caused you the greatest concern?	The number of notified cases	335	44.1
		The type of disease	223	29.3
		The fact that it happened at school	340	44.7
		The fear of being affected by tuberculosis	275	36.2
		Other fears	32	4.2
		Missing	19	2.5
13	Which sources of information did you preferentially use to find out about the disease and its possible consequences?	Institutional websites (Ministry of Health, Epicentro website of the National Health Institute)	398	52.4
		Search on specialized websites	119	15.7
		Social networks	44	5.8
		Television and newspapers	127	16.7
		Healthcare staff of local health authority	270	35.5
		General practitioner and pediatrician	192	25.3
		Other healthcare professionals	86	11.3
		Other people	77	10.1
		Missing	34	4.5

**Table 2:** Responses given by school staff, risk perception questionnaire *n* = 63.

	Question	Answer	*n*	%
1	Gender	Female	57	90.5
		Male	6	9.5
2	Age	≤30 yrs	6	9.5
		31–40 yrs	8	12.7
		41–50 yrs	14	22.2
		>50 yrs	35	55.6
3	Occupation	Teacher	48	76.2
		Auxiliary staff	15	23.8
4	In which grade do you teach or carry out activities?	1st grade	17	27.0
		2nd grade	13	20.6
		3rd grade	15	23.8
		4th grade	14	22.2
		5th grade	15	23.8
5	How many hours per week did you spend in close contact with the primary tuberculosis case?	None	41	65.1
		<1	17	27.0
		1–5	0	0
		6–10	0	0
		>10	0	0
		Missing	5	7.9
6	Did you know about tuberculosis as a disease before the recent events occurred at school?	Yes	55	87.3
		No	8	12.7
7	Were you aware of tuberculosis cases in your province of residence in the last 2 years?	Yes	19	30.2
		No	44	69.8
8	What was your perception of risk when you became aware of these tuberculosis cases?	No risk	5	7.9
		Low risk	22	34.9
		Moderate risk	21	33.3
		High risk	15	23.8
9	What is your current perception of risk also considering the intervention carried out by the LHA?	No risk	11	17.5
		Low risk	30	47.6
		Moderate risk	17	27.0
		High risk	5	7.9
10	Do you think the intervention of the LHA following notification of the first case of tuberculosis was timely?	Yes	57	90.5
		No	5	7.9
		Missing	1	1.6
11	Were the meetings with the LHA healthcare staff useful in clearly defining the situation?	Yes	53	84.1
		No	9	14.3
		Missing	1	1.6
11.1	If you answered No to the previous question, what do you think was missing?	I wanted more meetings	0	0
		Conflicting explanations were given	6	66.7
		The meetings were delayed	1	11.1
		Communication was not transparent	7	77.8
		The risk was underestimated	2	22.2
		Other	1	11.1
12	What aspect of the current tuberculosis outbreak caused you the greatest concern?	The number of notified cases	39	61.9
		The type of disease	10	15.9
		The fact that it happened at school	16	25.4
		The fear of being affected by tuberculosis	14	22.2
		Other fears	4	6.3
13	Which sources of information did you preferentially use to find out about the disease and its possible consequences?	Institutional websites (Ministry of Health, Epicentro website of the National Health Institute)	24	38.1
		Search on specialized websites	9	14.3
		Social networks	1	1.6
		Television and newspapers	7	11.1
		Healthcare staff of local health authority	33	52.4
		General practitioner and pediatrician	25	39.7
		Other healthcare professionals	4	6.3
		Other people	1	1.6

**Table 3:** Final results of logistic regression, binary risk among parents who perceived a moderate and high risk at T0. Log-likelihood = –286.451(OR = odds ratio; adj = adjusted; CI confidence interval; LR-test = likelihood ratio test; AIC value = Akaike’s Information Criterion value)

Included Variables	Crude OR (95%CI)	adj. OR (95%CI)	p (Wald’s test)	p (LR-test)
Meetings with healthcare staff of the local health authorities perceived as useful	1.95 (1.3,2.94)	2.08 (1.37,3.17)	<0.001	<0.001
Social networks as major source of information about the disease:	2.74 (1.04,7.25)	2.51 (0.94,6.73)	0.067	0.046
Gender: F vs. M	0.88 (0.6,1.3)	0.82 (0.55,1.22)	0.326	0.326
Educational level: ref. = University degree and Ph.D.				0.002
Primary school	0.13 (0.03,0.65)	0.12 (0.02,0.6)	0.01	
Lower secondary school	1.74 (0.92,3.28)	1.81 (0.95,3.46)	0.072	
Upper secondary school	1.12 (0.68,1.82)	1.23 (0.74,2.03)	0.419	

No. of observations = 483, AIC value = 568.902.

## Supplementary Materials

The following are available online at https://www.mdpi.com/1660-4601/17/3/911/s1, S1: Questionnaire design; S2, S3: Sign test of matched pairs and Wilcoxon matched-pairs signed-ranks test for parents and school staff.

## References

[1] WHO Expert Consultation on Outbreak Communications (2004: Singapore) & World Health Organization (2004). Communicable Diseases Cluster. 2005. Outbreak Communication: Best Practices for Communicating with the Public during an Outbreak: Report of the WHO Expert Consultation on Outbreak Communications held in Singapore, 21–23 September 2004. https://apps.who.int/iris/handle/10665/69138.

[2] Rubin G.J., Amlôt R., Pege L., Wessely S. (2009). Public perceptions, anxiety, and behaviour change in relation to the swine flu outbreak: Cross sectional telephone survey. BMJ.

[3] Smith R.D. (2006). Responding to global infectious disease outbreaks: Lessons from SARS on the role of risk perception, communication and management. Soc. Sci. Med..

[4] Seeger M.W., Pechta L.E., Price S.M., Lubell K.M., Rose D.A., Sapru S., Chansky M.C., Smith B.J. (2018). A Conceptual Model for Evaluating Emergency Risk Communication in Public Health. Health Secur..

[5] Tumpey A.J., Daigle D., Nowak G. Communicating During an Outbreak or Public Health Investigation, CDC, Epidemic Intelligence Service. https://www.cdc.gov/eis/field-epi-manual/chapters/Communicating-Investigation.html.

[6] Dettori M., Arru B., Azara A., Piana A., Mariotti G., Camerada M.V., Stefanelli P., Rezza G., Castiglia P. (2018). In the Digital Era, Is Community Outrage a Feasible Proxy Indicator of Emotional Epidemiology? The Case of Meningococcal Disease in Sardinia, Italy. Int. J. Environ. Res. Public Health.

[7] Dettori M., Azara A., Loria E., Piana A., Masia M.D., Palmieri A., Cossu A., Castiglia P. (2019). Population Distrust of Drinking Water Safety. Community Outrage Analysis, Prediction and Management. Int. J. Environ. Res. Public Health.

[8] Covello V.T. (1992). Risk Communication: An Emerging Area of Health Communication Research. Ann. Int. Commun. Assoc..

[9] McComas K.A. (2006). Defining Moments in Risk Communication Research: 1996–2005. J. Health Commun..

[10] Palenchar M.J., Heath R.L. (2007). Strategic risk communication: Adding value to society. Public Relat. Rev..

[11] Coombs W.T. (2018). Ongoing Crisis Communication: Planning, Managing, and Responding.

[12] Fischhoff B. (1995). Risk Perception and Communication Unplugged: Twenty Years of Process. Risk Anal..

[13] Centers for Disease Control and Prevention (2018). Crisis and Emergency Risk Communication: 2014 Edition.

[14] Coombs W.T., Holladay S.J. (2012). The Handbook of Crisis Communication.

[15] Freberg K., Palenchar M.J., Veil S.R. (2013). Managing and sharing H1N1 crisis information using social media bookmarking services. Public Relat. Rev..

[16] Brashers D.E. (2001). Communication and uncertainty management. J. Commun..

[17] Cinquetti S., Dalmanzio M., Ros E., Genrili D., Ramigni M., Grossi A., Andrianou X.D., La Torre L.E., Rigoli R., Scotton P.G. (2019). High rate of transmission in a pulmonary tuberculosis outbreak in a primary school, north-eastern Italy, 2019. Eurosurveillance.

[18] Ministero della Salute (2010). Aggiornamento Delle Raccomandazioni per le Attività di Controllo Della Tubercolosi. http://www.salute.gov.it/imgs/C_17_pubblicazioni_1661_allegato.pdf.

[19] IRCCS Spallanzani, Protocollo di Gestione Clinica Della Tubercolosi. http://www.inmi.it/wp-content/uploads/2018/12/Protocollo-Tubercolosi-Rev.-7_2017.pdf.

[20] ECDC, European Union Standards for Tuberculosis Care-2017 Update. https://www.ecdc.europa.eu/en/publications-data/european-union-standards-tuberculosis-care-2017-update.

[21] World Health Organization (2018). Communicating Risk in Public Health Emergencies: A WHO Guideline for Emergency Risk Communication (ERC) Policy and Practice. http://www.who.int/risk-communication/guidance/download/en/.

[22] Reynolds B.J., Seeger M.W. (2005). Crisis and Emergency Risk Communication as an Integrative Model. J. Health Commun..

[23] Reynolds B.J. (2011). When the facts are just not enough: Credibly communicating about risk is riskier when emotions run high and time is short. Toxicol. Appl. Pharmacol..

[24] Ryan M.J., Giles-Vernich T., Graham J.E. (2019). Technologies of trust in epidemic response: Openness, reflexivity and accountability during the 2014-2016 Ebola outbreak in West Africa. BMJ Glob. Health.

[25] Kinsman J. (2012). “A time of fear”: Local, national, and international responses to a large Ebola outbreak in Uganda. Glob. Health.

[26] Korda H., Itani Z. (2013). Harnessing social media for health promotion and behaviour change. Health Promot. Pract..

[27] Biswas M. (2013). Health organizations’ use of social media tools during a pandemic situation: An H1N1 flu context. J. New Commun. Res..

[28] World Health Organization (2012). Developing Tools for Strategic Communication to the Media on Emerging Infectious Diseases (EIDs). http://apps.searo.who.int/PDS_DOCS/B4803.pdf.

[29] Toppenberg-Pejcic D., Noyes J., Allen T., Alexander N., Vanderford M., Gamhewage G. (2019). Emergency Risk Communication: Lessons Learned from a Rapid Review of Recent Gray Literature on Ebola, Zika, and Yellow Fever. Health Commun..

[30] Jin Y., Austin L., Vijaykumar S., Jun H., Nowak G. (2019). Communicating about infectious disease threats: Insights from public health information officers. Public Relat. Rev..

[31] ECDC (2013). A Literature Review on Effective Risk Communication for the Prevention and Control of Communicable Diseases in Europe Insights into Health Communication. https://op.europa.eu/en/publication-detail/-/publication/f9d611d1-2acf-4c77-9dc1-b1e204901f34/language-en.

