# Supplementary material for: Impact of Communication Measures Implemented During a School Tuberculosis Outbreak on Risk Perception among Parents and School Staff, Italy, 2019
Source: Int J Environ Res Public Health. 2020 Feb 1;17(3):911. doi: 10.3390/ijerph17030911 (PMC7037209; doi:10.3390/ijerph17030911)
Supplement: Supplementary file 1 [file ijerph-17-00911-s001.pdf]

Supplementary Material

Supplementary S1

SURVEY PARENTS

1) Gender
□ Female □ Male

2) Age
□ ≤ 30 yrs □ 31-40 yrs □ 41-50 yrs □ > 50 yrs

3) Educational level
□ Primary school □ Lower secondary school □ Upper secondary school □ Higher education (degree and Ph.D)

4) Which grade does your child attend?
□ 1st grade □ 2nd grade □ 3rd grade □ 4th grade □ 5th grade

5) How many hours per week did your child spend in close contact with the primary tuberculosis case?
□ None □ Less than one □ From one to five □ From six to ten □ More than ten

6) Did you know about tuberculosis as a disease before the recent events occurred at school?
□ Yes □ No

7) Were you aware of tuberculosis cases in your province of residence in the last 2 years?
□ Yes □ No

8) What was your perception of risk when you became aware of these tuberculosis cases?
□ No risk □ Low risk □ Moderate risk □ High risk

9) What is your current perception of risk also considering the interventions carried out by the LHA?
□ No risk □ Low risk □ Moderate risk □ High risk

10) Do you think the intervention of the LHA following the notification of the first case of tuberculosis was timely?
□ Yes □ No

11) Were the meetings with the LHA operators useful in clearly defining the situation?
□ Yes □ No

11.1) If you answered No to the previous question, what do you think was missing?
□ I wanted more meetings □ Conflicting explanations were given □ The meetings were delayed
□ Communication was not transparent □ The risk was underestimated □ Other

12) What aspect of the current tuberculosis outbreak caused you the greatest concern?
□ The number of notified cases □ The type of disease
□ The fact that it happened at school □ The fear of being affected by tuberculosis □ Other fears

12.1) If you answered Other fears, specify your greatest concern ________________________________

13) Which sources of information did you preferentially use to find out about the disease and its possible consequences?

□ Institutional websites (Ministry of Health, Epicentro website of the National Health Institute)
□ Search on specialized websites □ Social networks □ Television and newspapers
□ Healthcare staff of Local Health Authority □ General practitioner and pediatrician
□ Other healthcare professional □ Other people

SURVEY SCHOOL STAFF

1) Gender
□ Female □ Male

2) Age
□ ≤ 30 yrs □ 31-40 yrs □ 41-50 yrs □ > 50 yrs

3) Occupation
□ Teacher □ Auxiliary staff

4) In which grade do you teach or carry out activities?
□ 1st grade □ 2nd grade □ 3rd grade □ 4th grade □ 5th grade

5) How many hours per week did you spend in close contact with the primary tuberculosis case?
□ None □ Less than one □ From one to five □ From six to ten □ More than ten

6) Did you know about tuberculosis as a disease before the recent events occurred at school?
□ Yes □ No

7) Were you aware of tuberculosis cases in your province of residence in the last 2 years?
□ Yes □ No

8) What was your perception of risk when you became aware of these tuberculosis cases?
□ No risk □ Low risk □ Moderate risk □ High risk

9) What is your current perception of risk also considering the intervention carried out by the LHA?
□ No risk □ Low risk □ Moderate risk □ High risk

10) Do you think the intervention of the LHA following the notification of the first case of tuberculosis was timely?
□ Yes □ No

11) Were the meetings with the LHA operators useful in clearly defining the situation?
□ Yes □ No

11.1) If you answered No to the previous question, what do you think was missing?
□ I wanted more meetings □ Conflicting explanations were given □ The meetings were delayed
□ Communication was not transparent □ The risk was underestimated □ Other

12) What aspect of the current tuberculosis outbreak caused you the greatest concern?
□ The number of notified cases □ The type of disease
□ The fact that it happened at school □ The fear of being affected by tuberculosis □ Other fears

12.1) If you answered Other fears, specify your greatest concern ________________________________

13) Which sources of information did you preferentially use to find out about the disease and its possible consequences?
□ Institutional websites (Ministry of Health, Epicentro website of the National Health Institute)
□ Search on specialized websites □ Social networks □ Television and newspapers
□ Healthcare staff of Local Health Authority □ General practitioner and pediatrician
□ Other healthcare professional □ Other people

Table S2: Sign test of matched pairs and Wilcoxon matched-pairs signed-ranks test for parents

Sign	Observed, n	Expected, n	Sum ranks, n	Expected, n
positive	375	231.5	192583	118528
negative	88	231.5	44473	118528
zero	280	280	39340	39340
all	743	743	276396	276396

Sign test

Two-sided test:
Ho: median of risk_perceptionT0 – risk_perceptionT1 = 0 vs.
Ha: median of risk_perceptionT0 – risk_perceptionT1 != 0
Pr(#positive >= 375 or #negative >= 375) =
min(1, 2*Binomial(n = 463, x >= 375, p = 0.5)) < 0.0001

Sign rank

unadjusted variance 34250071
adjustment for ties -853220.5
adjustment for zeros -1839145
adjusted variance 31557706

Ho: risk_perceptionT0 = risk_perceptionT1
z = 13.183
Prob > |z| < 0.0001

Table S3: Sign test of matched pairs and Wilcoxon matched-pairs signed-ranks test for school staff

Sign	Observed, n	Expected, n	Sum ranks, n	Expected, n
positive	27	15.5	1310	744
negative	4	15.5	178	744
zero	32	32	528	528
all	63	63	2016	2016

Sign test

Two-sided test:
Ho: median of risk_perceptionT0 – risk_perceptionT1 = 0 vs.
Ha: median of risk_perceptionT0 – risk_perceptionT1!= 0
Pr(#positive >= 27 or #negative >= 27) =
min(1, 2*Binomial(n = 31, x >= 27, p = 0.5)) < 0.0001

Sign rank

unadjusted variance	21336.00
adjustment for ties	-294.50
adjustment for zeros	-2860.00
adjusted variance	18181.50

Ho: risk_perceptionT0 = risk_perceptionT1

z = 4.198

Prob > |z| < 0.0001
